# Maxillary gingival neurolemmoma: a case report and literature review

**DOI:** 10.1186/s12903-023-03509-7

**Published:** 2023-10-19

**Authors:** Xiangzi Zhang, Qiming Gao, Yunze Xuan

**Affiliations:** 1 Yanbian University Medical College, JiLin, 133000 China; 2 Stomatology Department, Affiliated Hospital of Yanbian University, JiLin, 133000 China

**Keywords:** Neurolemmoma, Maxillary gingiva, Intraoral lesion, Clinical characteristics

## Abstract

**Objective:**

To explore and summarize the clinical features, differential diagnosis and treatment of the oral maxillofacial schwandoma.

**Case presentation:**

This is a report of a case of a 46-year-old female patients with neurolemmoma in the maxillary gingiva. The clinical features, pathological features, differential diagnosis and treatment were analyzed. Literature review was conducted in search of domestic and overseas journal full-text database from 1986 ~ 2017. 39 reports on the oral and maxillofacial Neurolemmoma from 1986 to 2017 in the database of China hospital knowledge database and the PubMed database, there were 405 patients. There were 23 cases of gingival mucosa, 17 in foreign literature and only 6 in the domestic literature.

**Conclusions:**

The incidence of gingival Neurolemmoma is extremely low, the predilection age is similar to other parts, it is middle-aged and young, and there is no obvious gender tendency. About 25–45% of schwannomas are found in the head and neck, and rarely in the mouth (only 1%). The most common internal location of the mouth is the tongue, followed by the floor of the mouth, buccal mucosa, palate, gums, and lips. Schwannomas are slow-growing benign tumors that are rare in the gums. Gingival schwannoma is usually a single occurrence, and the clinical manifestations are mostly painless gum mass, tooth loosening and displacement, without peripheral bone changes and regional lymph node metastasis. It is difficult to diagnose this tumor according to clinical manifestations, and pathological diagnosis is still the basis for the diagnosis of gingival schwannoma. So far, surgical resection is the preferred treatment for this disease, and the prognosis is good.

## Background

Neurolemmoma, also known as Schwannoma, is a benign nerve sheath tumor arising from perineural Schwann cells, which develop during the 4th week of gestation. Neurolemmoma grows slowly, mostly single, with intact envelope,and is a benign tumor, with occasional malignant changes [1].

For benign wrapping lesions, the best treatment option is to completely remove the entire tissue and preserve the surrounding healthy tissue. To date, there is no established drug treatment for schwannomas, and for painful schwannomas, gabapentin or pregabalin, short-acting opioids, or nonsteroidal anti-inflammatory drugs can successfully reduce pain in patients with schwannomas. Other medications, such as amitriptyline, duloxetine, topiramate, or carbamazepine, may be used as an adjunct or alone [2].

The clinical features, pathological features, differential diagnosis and treatment of maxillary gingival Neurolemmoma were analyzed based on a case of maxillary gingival Neurolemmoma admitted to our hospital and the literature of gingival Neurolemmoma reported in Chinese and foreign journals from 1986 to 2017.

## Case Report

**Fig. 1:**
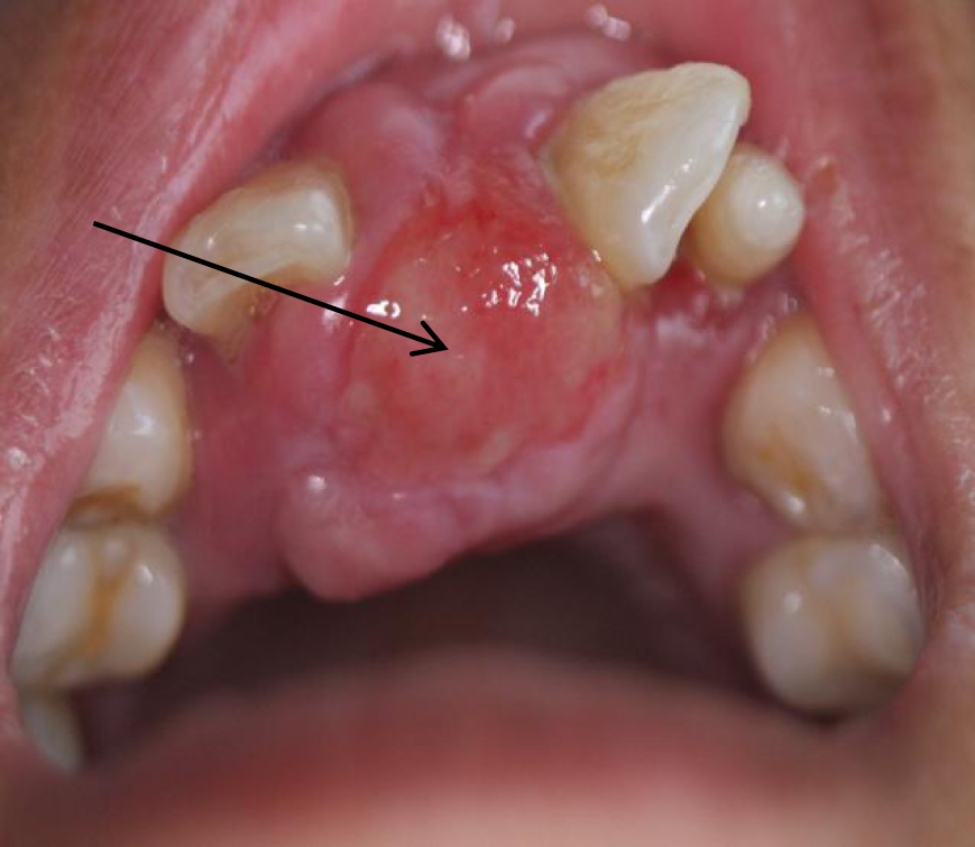
Macroscopic view in oral cavity

**Fig. 2:**
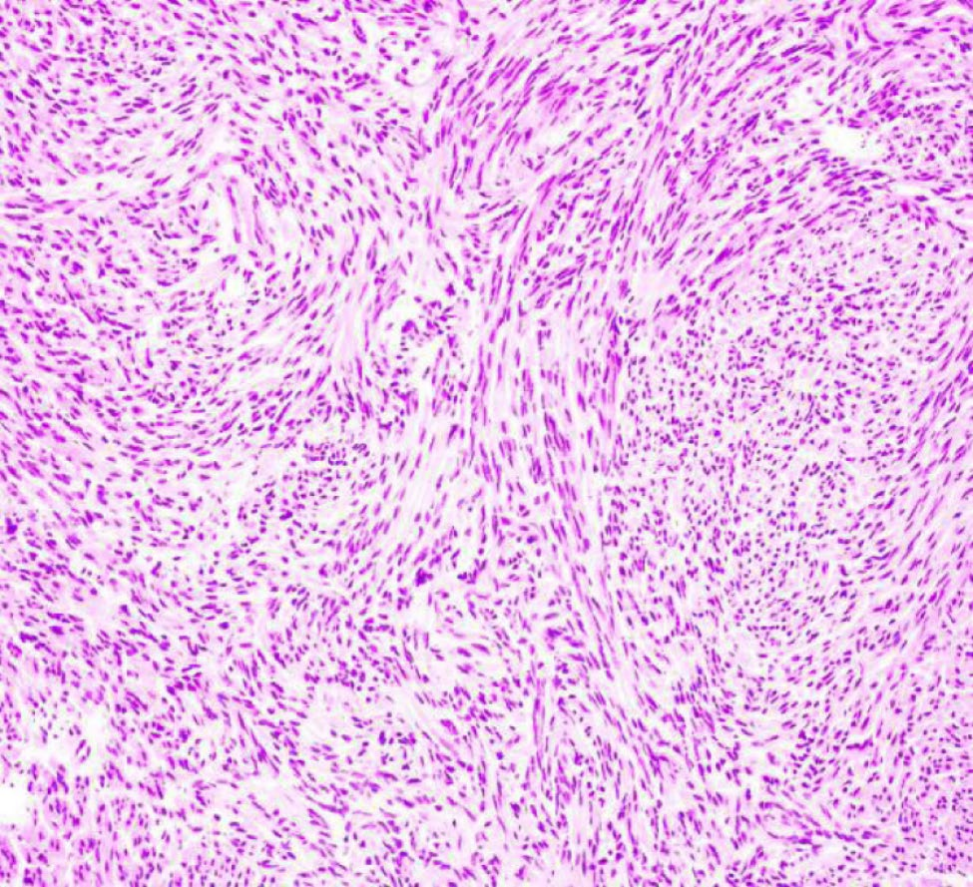
Pathological examination results

**Fig. 3:**
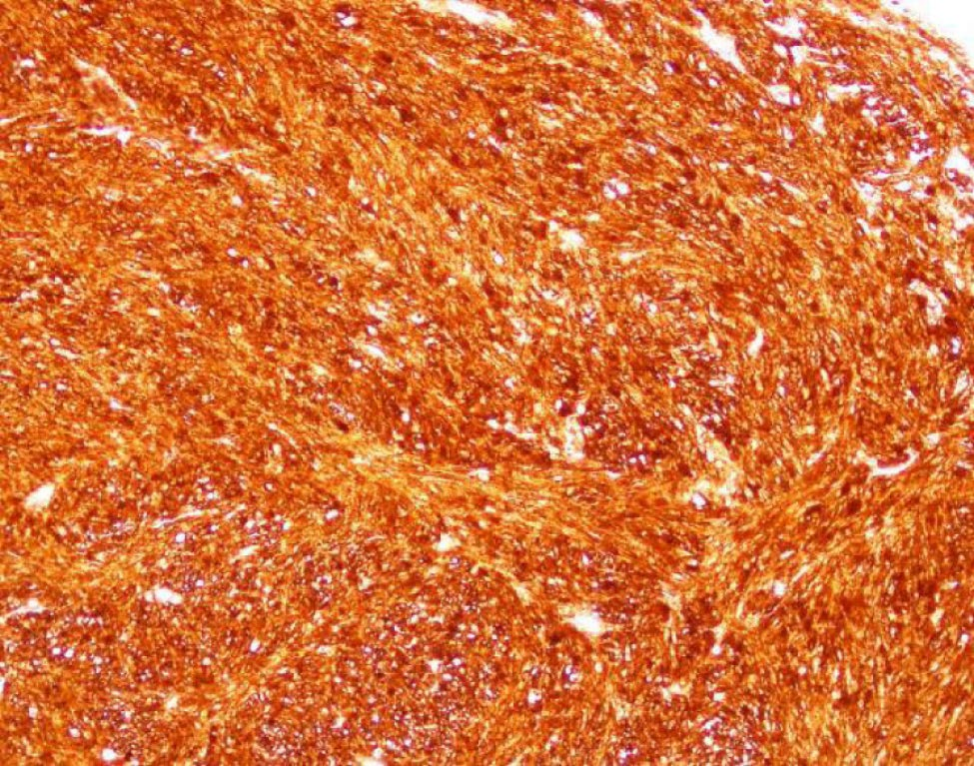
S−100, Immunohistochemistry, two-step method, ×20fold

**Fig. 4:**
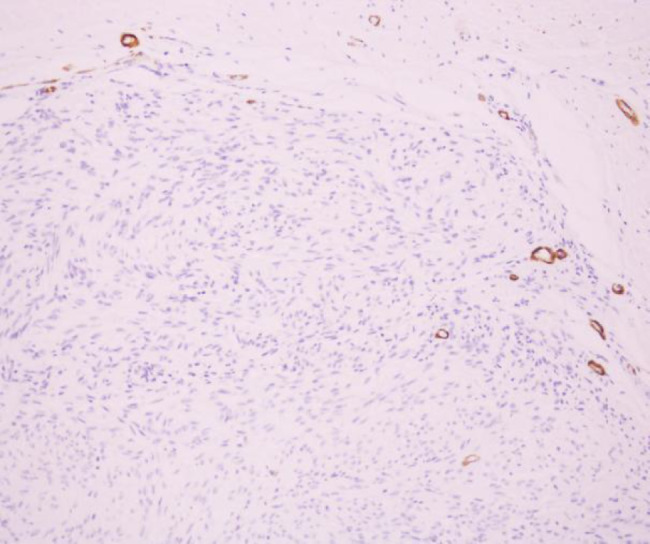
α-SMA, Immunohistochemistry, two-step method, × 20-fold

The patient was a 46-year-old female. The patient was admitted to the hospital because of recurrent gingival swelling in the upper anterior region for 20 years and recent aggravation of pain. Oral examination revealed a 3 cm × 2 cm × 1 cm mass in the gingival region between the maxillary central incisors, dark red in color, smooth surface, hard in texture, tender, and clear boundary with surrounding normal mucosal tissue. Distal gingival inclination, III. Loosening, percussion (±), II. Loose, its gingiva bleeding easily on probing, periodontal pocket 4 mm (Fig. 1). No obvious bone destruction was found in imaging examination. Clinical diagnosis: maxillary epulis. Pathological examination of local biopsy showed that the tumor cells were spindle-shaped, the nuclei were long and round, and densely arranged in a palisade shape. Immunohistochemical staining showed S-100 protein (+) and α-SMA (-) (Figs. 2, 3 and 4). The pathological diagnosis was Antoni type A Neurolemmoma of the gingiva. Treatment: complete resection of the tumor. There was no recurrence after 8 months of follow-up.

## Literature review

A total of 39 reports on oral and maxillofacial Neurolemmoma from 1986 to 2017 were retrieved from China hospital knowledge database and PubMed database, with a total of 405 patients, including 200 males, 204 females, and 1 case without gender. The age ranged from 3 months to 81 years. The lesions involved parotid gland, submandibular region, infraorbital region, upper neck, upper and lower jaw, nasal cavity and paranasal sinuses, chin, upper and lower lip, hard and soft palate, tongue, mouth floor mucosa, buccal mucosa, upper and lower gingiva, and lateral pharyngeal wall. Among them, 23 cases were reported involving the gingival mucosa, 17 cases were reported in foreign literature, and only 6 cases were reported in domestic literature (Table 1).

**Table 1:** Gingival Neurolemmomas published in domestic and foreign literatures from 1986 to 2017 (part)

Author	Birth	Gender	Age	Part
M.B Guglielmotti [8]	1987	male	18	Mandible gingival
Chrysomali et al. [31]	1997	unknown	unknown	Maxillary gingival
Fanghe,Pang [28]	1999	unknown	unknown	Gingiva in the upper and lower posterior teeth region
Lombardi et al. [32]	2002	unknown	unknown	Maxillary gingival
Huangfang [30]	2003	unknown	unknown	Maxillary gingival
Federica Demarosi [10]	2008	female	14	Mandible gingival
Ioannis G [7]	2010	unknown	unknown	Maxillary gingival
Parth Puwar [14]	2014	male	10	Maxillary gingival
Fumio Ide [19]	2015	female	35	Mandible gingival

## Discussion and conclusion

### Predilection site

Neurolemmoma was first reported by Verocay in 1908 [7]. It can occur in any part of the body, and the head and neck is one of the most common sites [3]. According to the latest literature, about 25–45% of Neurolemmomas occur in the head and neck, of which only 1–12% occur in the oral soft tissue [4]. Neurolemmoma in the anterior one-third of the tongue is the most common site of oral soft tissue [5], followed by palate (including hard and soft palate mucosa), mouth floor mucosa, buccal mucosa, lip and vestibular groove mucosa [6], while gingival Neurolemmoma is extremely rare in clinical practice. The tumor is more common in the maxillary gingiva than in the mandibular gingiva, and the occurrence ratio is 1:0.176. The reason may be that the distribution of nerves in the maxilla is more abundant than that in the mandible, and more nerves pass through the maxillary gingiva than the mandibular gingiva. The sample size investigated in this paper is small, and the results may be biased.

### Age and gender

Neurolemmoma can occur at any age, but it is most common in young and middle-aged people [3]. According to the analysis of a patient admitted to our hospital and 405 cases investigated in this paper, the youngest age of onset was 3 months, the highest was 81 years old, and the high incidence range was 20–40 years old, which was similar to the results reported in domestic and foreign literatures. There are various opinions on the prevalence gender of this disease in domestic and foreign literature. William et al. believe that this tumor is more likely to occur in males [9], Federica Demarosi and Peng Lingling et al. believe that this tumor is more likely to occur in females [10, 11]. Monir Moradzadeh, Khiavi et al., and most scholars believe that Neurolemmoma in the oral and maxillofacial region has no obvious gender susceptibility [12]. According to the analysis of one case admitted to our hospital and 405 cases investigated in this paper, after excluding one case whose sex was unknown, there were 200 male patients and 205 female patients. The ratio of male to female was 1:1.025, and there was no obvious gender susceptibility, which was the same as most of the literature. The ratio of male to female was 1.1:1.2. The female was slightly more susceptible than the male.

### Clinical features

Neurolemmoma often occurs alone. Ge Shufen et al. found 2 cases of multiple cases in the same part of the oral and maxillofacial region and 1 series of multiple cases in the whole body [13], which are rare. Parth Puwar et al. reported a case of maxillary gingival Neurolemmoma with complete gingival hyperplasia [14].

Neurolemmoma is usually painless and grows slowly until it reaches a certain size, and aesthetic and functional disorders appear according to different parts [15]. Neurolemmoma is usually a solid mass with smooth surface, clear boundary and no adhesion to the surrounding tissues. However, with the increase of the tumor, surface ulceration, bleeding and erosion may occur. Liquefaction necrosis and cystic degeneration may occur in the parenchyma, and the possibility of intratumoral hemorrhage should be considered in Neurolemmoma with sudden enlargement [16]. When the tumor becomes larger, it often compresses the surrounding bone wall to make it absorb and soften, but it will not invade the surrounding tissues [17]. According to the investigation in this paper, no surrounding bone changes (including malignant gingival Neurolemmoma) were found in 23 cases of gingival Neurolemmoma.

### Pathological diagnosis and differential diagnosis were made

The clinical manifestations of gingival schwannoma are non-specific, and the diagnosis is difficult. Pathological examination is necessary. S-100 protein and human natural killer-1 can be used for immunohistochemical examination, but they are not specific. Glial fibrillary acidic protein labeling is mostly positive, which is of great significance [18]. Histopathologically, schwannomas are classified into AntoniA type and AntoniB type. Antoni type A was characterized by vortex-like arrangement of tumor cells with long rod-shaped nuclei arranged in a palisade pattern. Numerous blood vessels, reticular fibers, and collagen fibers could be seen in the tumor. Antoni type B is characterized by sparse and messy tumor cells, mostly dysplasia or fatty changes, and more reticular fibers or collagen fibers in the stroma [19]. Huang Fang et al. believed that the two types usually coexist in the same tumor and continue with each other. Antoni type A is the main type for small tumors, and Antoni type B is the main type for degeneration when the tumors are large [20].

Gingival schwannoma should be differentiated from other benign tumors and chronic inflammation in the primary site, such as epulis, lipoma, gingival hyperplasia, central and peripheral giant cell granuloma [21].

### Treatment and prognosis

The treatment of gingival schwannoma is the same as that of schwannoma occurring in other parts. Anti-inflammatory treatment is ineffective [22], and surgical resection is the preferred treatment [23]. So far, no case of benign schwannoma with regional lymph node metastasis or distant metastasis has been found, and the recurrence rate after complete resection is very low, and the prognosis is good [24]. However, malignant schwannoma has a high recurrence rate and poor prognosis, with a 2-year survival rate of only 45–70% [25]. Therefore, postoperative radiotherapy and chemotherapy should be combined with malignant schwannoma to prevent recurrence and distant metastasis [26–29].

## Data Availability

Data sharing is not applicable to this article as no datasets were generated or analysed during the current study.
